# Real-time neural network based predictor for cov19 virus spread

**DOI:** 10.1371/journal.pone.0243189

**Published:** 2020-12-17

**Authors:** Michał Wieczorek, Jakub Siłka, Dawid Połap, Marcin Woźniak, Robertas Damaševičius

**Affiliations:** 1 Faculty of Applied Mathematics, Silesian University of Technology, Gliwice, Poland; 2 Department of Applied Informatics, Vytautas Magnus University, Kaunas, Lithuania; Newcastle University, UNITED KINGDOM

## Abstract

Since the epidemic outbreak in early months of 2020 the spread of COVID-19 has grown rapidly in most countries and regions across the World. Because of that, SARS-CoV-2 was declared as a Public Health Emergency of International Concern (PHEIC) on January 30, 2020, by The World Health Organization (WHO). That’s why many scientists are working on new methods to reduce further growth of new cases and, by intelligent patients allocation, reduce number of patients per doctor, what can lead to more successful treatments. However to properly manage the COVID-19 spread there is a need for real-time prediction models which can reliably support various decisions both at national and international level. The problem in developing such system is the lack of general knowledge how the virus spreads and what would be the number of cases each day. Therefore prediction model must be able to conclude the situation from past data in the way that results will show a future trend and will possibly closely relate to the real numbers. In our opinion Artificial Intelligence gives a possibility to do it. In this article we present a model which can work as a part of an online system as a real-time predictor to help in estimation of COVID-19 spread. This prediction model is developed using Artificial Neural Networks (ANN) to estimate the future situation by the use of geo-location and numerical data from past 2 weeks. The results of our model are confirmed by comparing them with real data and, during our research the model was correctly predicting the trend and very closely matching the numbers of new cases in each day.

## 1 Introduction

Situation of world cov19 epidemia is very dynamic, and without models of prediction we are not able to estimate how the situation will develop. The problem in construction of such advisory systems is the lack of knowledge and data to compose them easily since we dont have any information about the virus spread before it start. We can estimate it just by the data from an extremely short period of time in which the situation changes rapidly. There are several methods which help to make prediction in such situations, however mostly reported are neural networks due to good generalization and precise prediction in case of uncertain environments.

In engineering neural networks work as predictors from micro-array data [13] and in photo-voltaic systems performance [14]. We can also find many other applications in real life, where we can base infrastructure decision about road accidents [15] or evaluate ecologic influence from ozone concentration [16]. Neural network predictors are also good in social modeling to predict suicide deaths [17].

In medicine neural networks are also very good predictors of new cases or progression of the disease. In [18] was presented how to model an ad-hoc predictor of arrhythmia from ambulatory electrocardiograms, which can support urgent decisions saving life. In medical systems data from various sensors can serve as knowledge for prediction models. Health sensoric data can be used to predict kidney diseases as shown in [19], heart failure as discussed in [20] or mRNA engineering from alternative polyadenylation [21]. Deep learning structure serves as pulmonary changes detection from cov19 virus as discussed by [22] and [23]. It is also possible to use neural networks as remote processing controls in cloud teleophthalmology IoT system as proposed in [24]. Some of models also give explainable premises of the disease symptoms as decision support model [25] or inflammation in Crohn’s disease [26]. A wide survey on positive aspects of using machine learning techniques to predict medical symptoms from diabets was presented in [27]. Artificial Intelligence also serves as death or survival predictor. In many cases neural networks were reported to succeed in estimating situation of patients or future spread of some diseases which lead to fatal death of people. In [28] was described a model developed for predicting in-hospital mortality of patients with kidney injury, while in [29] model for survival of breast cancer was developed. In [30] neural networks were described to predict clinical events in Intensive Care Unit. Neural networks also can predict future spread of respiratory disease. In [31] was discussed how Artificial Intelligence can be used to predict infection from inhale of Pseudomonas aeruginosa in Intensive Care Unit. In recent time many works are oriented on computation for cov19 virus spread both in regional and world range. In Table 1 is presented a summary of recent approaches. From the table we can learn that various models have been used to predict the spread in local and global aspects. However each of these analysis is using mathematical solutions based on calculations. Our proposed approach is oriented on machine learning (especially on devoted neural network architecture) and because of this proposed system is able to predict the cov19 spread trend for various countries and regions in any place on the Globe it makes a big advantage for our proposal.

**Table 1:** Summary of various models for cov19 prediction and outcome analysis in recent reports from research and statistics.

Authors	Method	Reach
Wu et al. [1]	spread of cov19 from the origin in the Wuhan city in China to other parts of country and further by using Markov Chain Monte Carlo method	Wuhan region, China
Zhang et al. [2]	turning point analysis by using Poisson process model	Major western countries
Gupta et al. [3]	spread of cov19 by using auto-regressive integrated moving average model	India
Fanelli et al. [4]	regional spread of cov19 by using iterative map of geometric sequence	Italy, France and China
Li et al. [5]	cov19 registered cases comparative analysis	China, South Korea, Iran and Italy
Pandey et al. [6]	analysis of registered and potential cases and regression model	India
Ivanov et al. [7]	impact of cov19 on economy by using anyLogistix simulation and optimization software	global
Gatto et al. [8]	cov19 spread by using metacommunity Susceptible–Exposed–Infected–Recovered (SEIR) transmission model	Italy
Petropoulos et al. [9]	cumulative data analysis	global
Anastassopoulou et al. [10]	cov19 spread by using Susceptible-Infectious-Recovered-Dead (SIDR) model	Hubei region, China
Bertozzi et al. [11]	data driven approach to prediction by analyzing number of cases	USA
Elmousalami et al. [12]	time series forecasting	global

In this paper we present an idea for the real-time prediction model based on neural networks. The system is using simple data available from governmental services to estimate the situation worldwide. The lack of general knowledge how the virus spreads and what would be the main reason for this make in not easy to predict the number of cases each day. Therefore prediction model must be able to conclude the situation from few past data information in the way that the results will show a reliable trend and will possibly closely relate to the real number of cases. Proposed prediction model is using neural networks to do it. At the beginning the data is normalized to help find relations between neighbor countries or regions and after that an input vector is presented to the nested neural network model in which we have composed devoted architectures to predict situation. First neural network is working on 12 days time window to predict the situation in the World, while second one is working with 8 days time window to predict the situation in regions of a country. The proposed model works well in both cases and is able to give a helpful estimation of the trend which can be used for planning but also closely approaches the real number of cases each day.

## 2 Neural prediction model

Let us now present the idea for the developed prediction model composition and training from the input data.

We use the dataset obtained from Johns Hopkins University Center for Systems Science and Engineering and available the on-line GitHub repository at https://github.com/datasets/covid-19. Simultaneously the data can be take from governmental services in each of countries. The dataset file is firstly pre-processed. Since we want the input data used by our neural network to be consistent, we have divided our data file into sections, using the following Algorithm Algorithm 1 for data pre-processing.

- The first section is the location name. It is not used in the training phase but it is used in the final step to create results table.
- The second section is geographical location given in longitude and latitude. It is used in the model training to specify which region has the biggest danger factor based on the neighboring countries and regions. The combination of geographical position is very useful for the system to predict how the number of confirmed cases may grow in countries or regions in relation to the surrounding ones.
- The third and final section contains 12 (in case of world model) and 8 (in case of region model) latest days of total confirmed cases for specified locations. Using such data the network can predict the approximate trend, which specifies how fast the virus will spread by showing the predicted number of cases in each of them.

**Fig 1:**
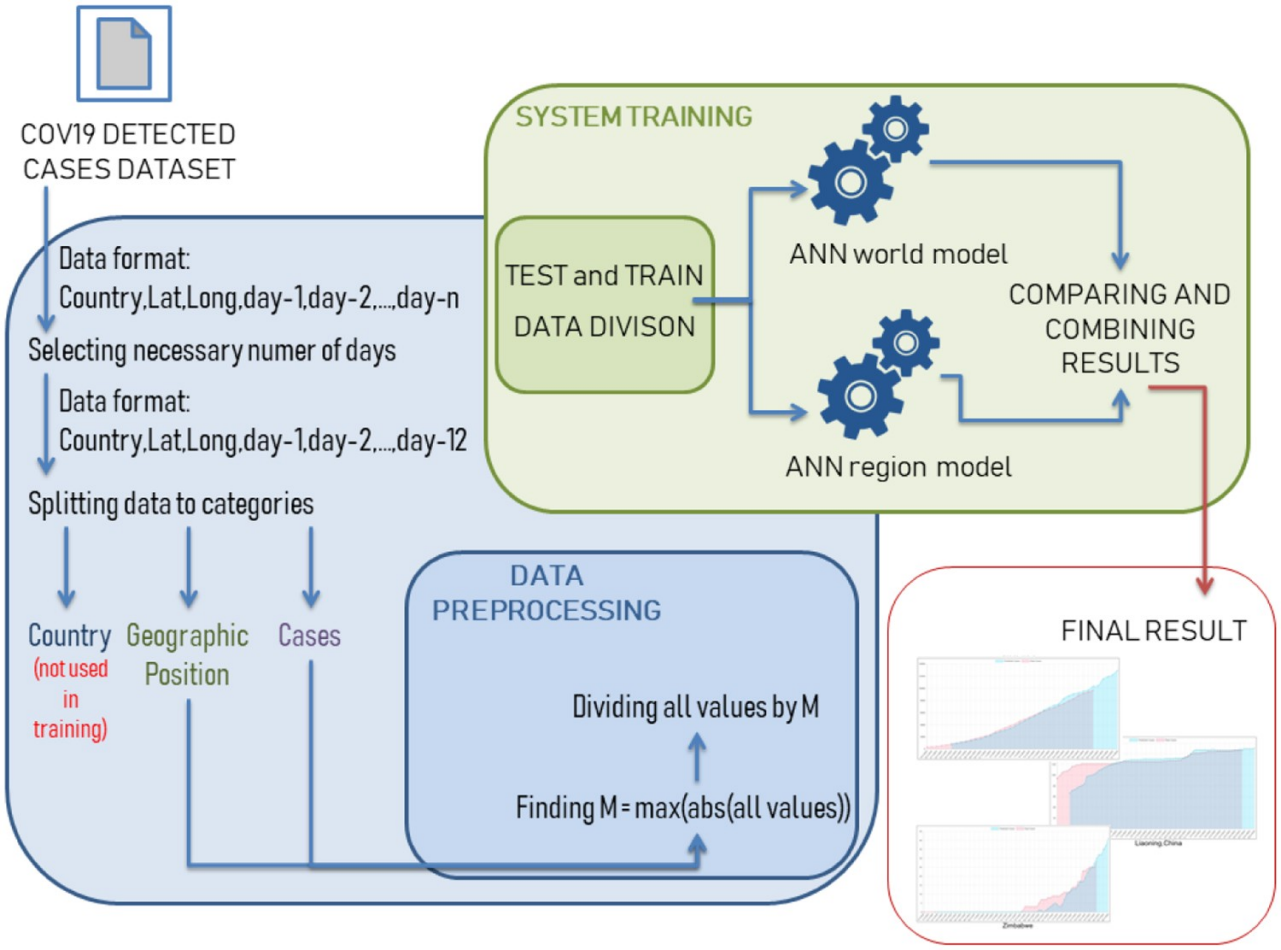
The system prediction model is divided into parts. Firstly we normalize input data to make the model more accurate for each of them. After, the data is forwarded to two neural network architectures. One, developed for the World prediction model, which is bigger due to wider spectrum. Second, developed for country regions prediction model, which is smaller.

Then the data before forwarding to the neural network is normalized. All numerical values are divided by the maximum value to normalize it to the range of values from 0 to 1. It helps to train the network and allows us to use the non-linear activation functions such as hyperbolic tangent. Schematic representation of the proposed prediction model is presented in Fig 1, which shows each stage of data processing from extraction to prediction.

**Algorithm 1** Data pre-processing algorithm

**Input**: Cov19 global confirmed cases dataset

1: split dataset to numerical and non-numerical values

2: region names are removed from training data and stored for later usage

3: numerical data are split to categories

4: geolocation is added to the training data

5: **for all** days **do**

6:  read date

7:  **if** date older than necessary number of days **then**

8:   do nothing

9:  **else**

10:   add day to the training data

11:  **end if**

12: **end for**

13: search for biggest value in training data

14: normalize it—divide all values by the biggest value

**output**: normalized and optimized training data for neural networks

### 2.1 Neural network

**Fig 2:**
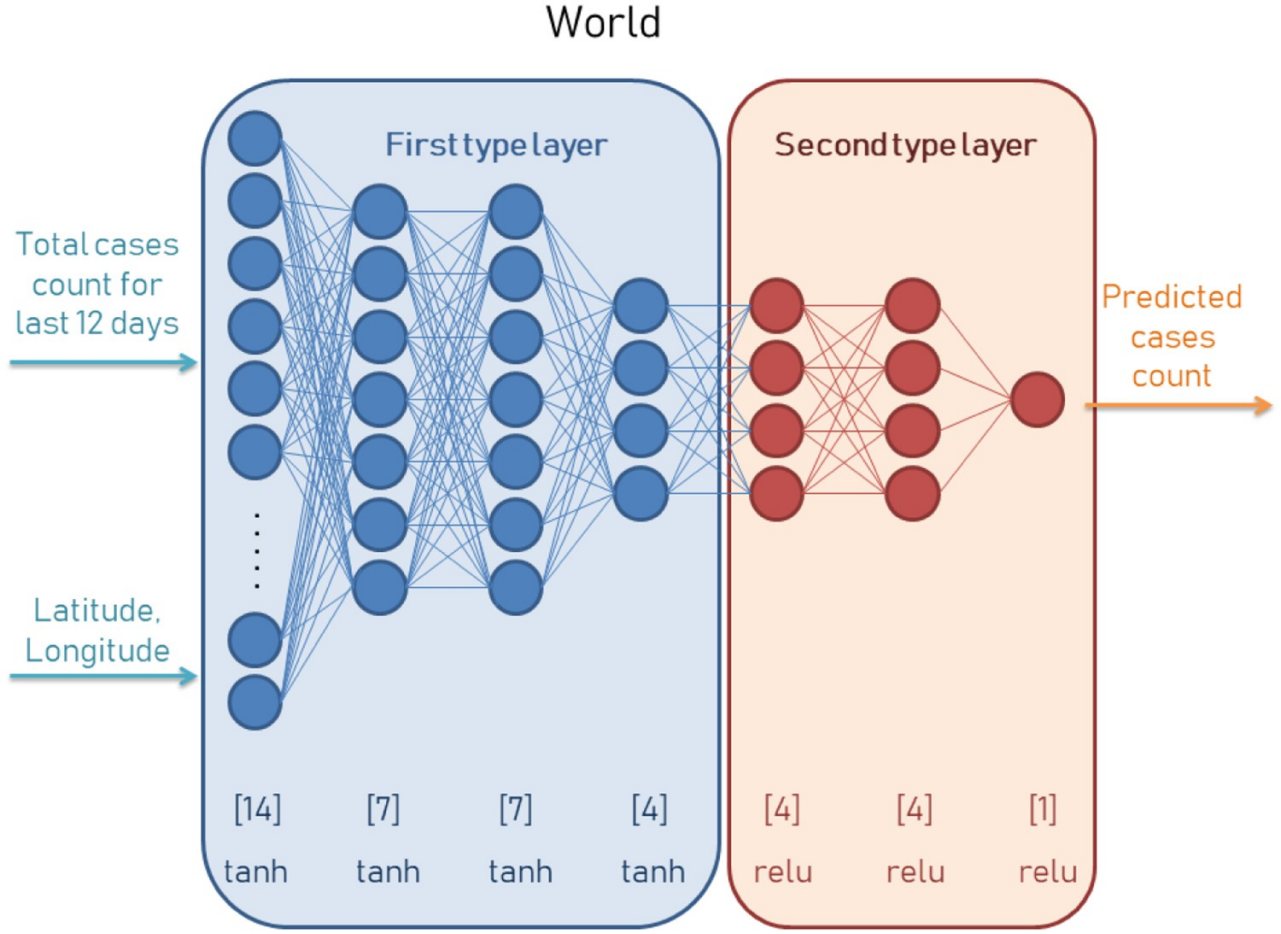
Neural network predictor for the number of cases in various world countries. The input data contains information from the last 12 days plus geolocation coordinates of latitude and longitude which have an impact on the cases correlations between neighboring countries.

**Fig 3:**
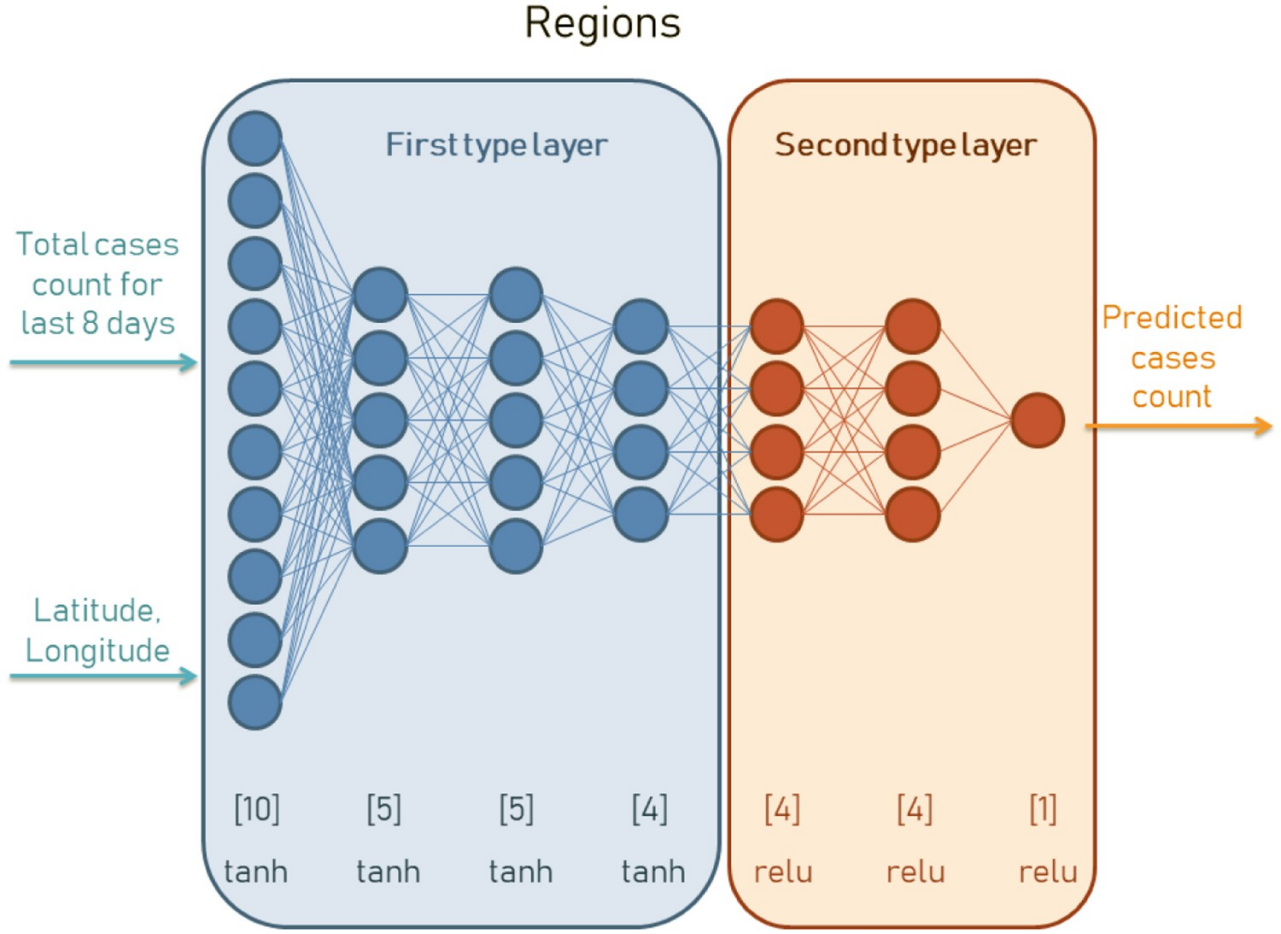
Neural network predictor for the number of cases in various regions of countries. The input data contains information from the last 8 days plus geolocation coordinates of latitude and longitude which have great impact on the cases correlation between neighbor regions. In case of regions in one country we have used shorter time window since in current situation when countries are isolated there is much higher daily correlation.

In the proposed predictor two separate neural networks cooperate on the input data processing to predict the growth of numbers and trend. Architecture one is bigger, since it is developed for the prediction of countries. In Fig 2 we can see how this architecture is composed. This neural network accepts the data from last 12 days along with geolocation in latitude and longitude. Being more specific, the day N prediction for the global model is based on days N-5 to N-17 and the day N prediction for the regional models are based on days N-5 to N-13. This information is processed on three hidden layers of first type and two hidden layers of second type. The number of neurons in each of them is selected empirically after tests we have done with the datasets used in our research. In Fig 3 we can see an architecture of neural network composed for regional prediction. This architecture is smaller as we can see in the number of neurons in first type layers. In both cases the neural network is implemented with two types of activation functions: hyperbolic tangent (for the first type layer) and relu function (for the second type layer).

**Algorithm 2** ADAM algorithm applied to train our prediction system

**Input:** *ϵ* = 0.001, *β*_1_ = 0.9, *β*_2_ = 0.999, *η* = 0.001

1: *t* = 0

2: **while** error is higher than 0.001 **do**

3:  Calculate momentum update with 1st momentum approach on *β*_1_ hyper-parameter (1)

4:  Calculate oscillations update with RMSprop approach on *β*_2_ hyper-parameter (2)

5:  Calculate corrections values (3) and (4)

6:  Correct weights on the neural network using (5)

7:  *t*++

8: **end while**

**Output**: trained neural network prediction system

### 2.2 System training

For the training of the neural networks, we have used an adaptive moment estimation algorithm called Adam [32]. It is the latest trend in research on usability of neural networks because of fast and not demanding processing ability. Adam algorithm is based on the first and second moments of gradients. To introduce this algorithm, it is necessary to provide basic formula for mean and variation. These coefficients in *t*-iteration are based on values in previous iteration marked as *t* − 1. Adam formula, as a combination of 1st momentum and RMSprop, can be described as follows

$$\begin{array}{c} m_{t}=\beta_{1}m_{t-1}+\left(1-\beta_{1}\right)g_{t}, \end{array}$$ (1)

$$\begin{array}{c} v_{t}=\beta_{2}v_{t-1}+\left(1-\beta_{2}\right)g_{t}^{2}, \end{array}$$ (2)

where *β* parameters are constant values called hyper-parameters and *g* is the current gradient value of error function for the neural network training. Values *m*_*t*_ and *t*_*m*_ are used for calculation of the correlations marked as ${\hat{m}}_{t}$ and ${\hat{v}}_{t}$ according to

$$\begin{array}{c} {\hat{m}}_{t}=\frac{m_{t}}{1-\beta_{1}^{t}} \end{array}$$ (3)

$$\begin{array}{c} {\hat{v}}_{t}=\frac{v_{t}}{1-\beta_{2}^{t}}. \end{array}$$ (4)

Using above calculated correlation of mean and variation, the final formula for changing weights in our neural network can be defined as a change between current weight *w*_*t*_ and calculated correlations

$$\begin{array}{c} w_{t+1}=w_{t}-\frac{\eta }{\sqrt{{\hat{v}}_{t}}+\epsilon }{\hat{m}}_{t}, \end{array}$$ (5)

where *η* is a learning rate and *ϵ* is a constant small value. The whole procedure is presented in Algorithm 2.

## 3 Results

Let us now discuss the results and efficiency of the proposed prediction model.

### 3.1 Finding the best data amount for our predictors

**Fig 4:**
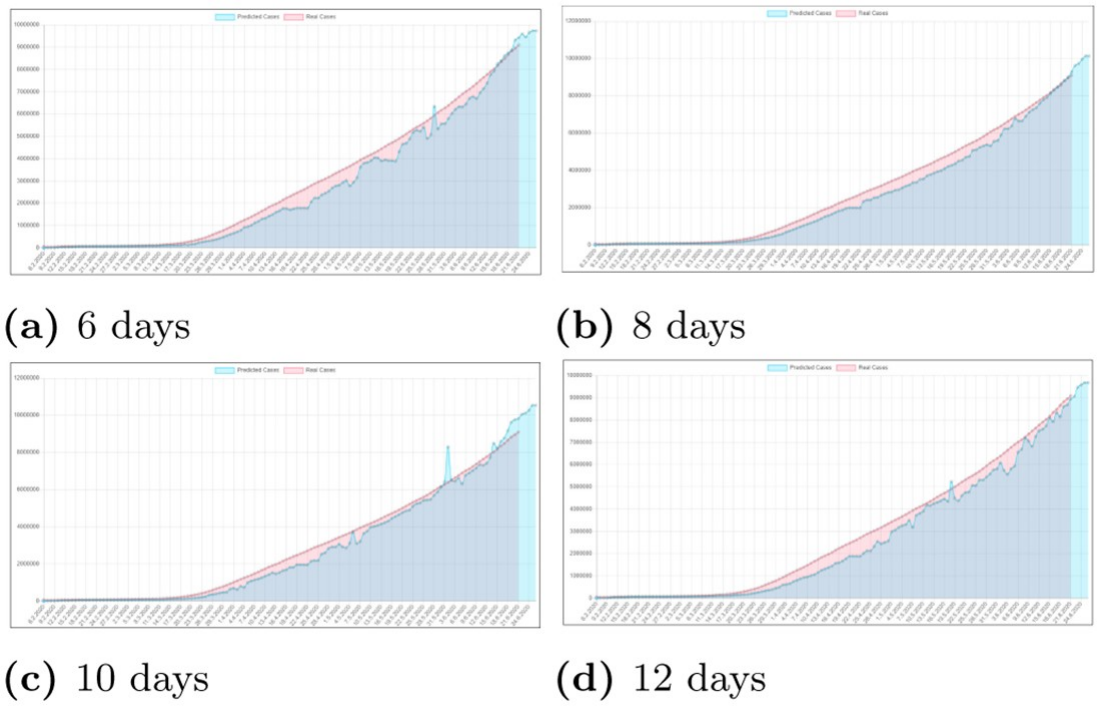
Results of changing input days amount for neural network predictor from 6 to 12 days.

In our research we have been searching for the number of days which should be given to the neural network for best prediction result. Because we started our research in a very early stage of global epidemia the dataset was very small and contained a very limited amount of days. Thus we wanted to reduce the amount of days needed to predict the future values to the minimum. That’s why we have done some experiments which results are shown in Fig 4. As we can see that using less days on the input resulted in lower stability of prediction with such data, although the main trend curve was correct. Going over 12 days, however, did not change a lot so we decided that 12 days for the global predictor would be the best point. The same thing was happening in the regions predictor, however because it worked on much smaller scale less days were needed and the best value was around 8 days.

### 3.2 Data normalization

Because each day our network is retrained to get the most accurate predictions, we normalize data in the way below:

- First we divide data into 2 sections: geo-localization values and total cases values,
- Next, we divide all total cases values by the maximum value from the dataset to get input values normalized from 0 to 1,
- Finally we normalize geo-localization data separately using min-max algorithm where min and max are defined by maximum and minimum latitude and longitude values.

Because we have used ReLu activation functions our generated predictions can exceed the maximum value from the training dataset, so after retraining our newly generated predictions are “denormalized” by multiplying all values by the maximum value of total cases we have divided before during normalization.

### 3.3 Test/train division

There are hundreds of variables defining COVID-19 spread speed and they vary in each country. So to correctly predict values for all countries we could not divide our dataset to test/train using the standard way, where we move some countries to test and the rest to training data because it would lead to more errors in the final results and the network would not fit well the curves of individual countries, however it should still perform well in the total cases sum of all countries.

Therefore we had to find another solution to test our model’s performance. To do this we divided our data to test/train not by countries but by days. Results from different period of training are shown in Fig 4. We have trained our model on data from 8 days before the newest date and older, and tested it on the newest data. Because of that our network could correctly adapt to every country separately and we could test if it generates the trend curve successfully or not. The only possible drawback of this approach is that our predictor network may have a little slip in the beginning of the epidemic period in some countries but after few days it adapts shortly giving us valid results.

In our experiment we tried to achieve the lowest possible error value for Mean Squared Error function. We did some experiments to see what lowest value of the error our proposed neural network can achieve. We were aiming to have the error at the level of about 0.01. At 4000 iterations, the network obtained the minimum of the loss function for our assumed level and then continued to decrease but actually without any spectacular changes. Therefore 4000 was experimentally considered the golden mean in the number of training iterations for our model.

### 3.4 Classic statistical approach

Statistical approach has been used to model prediction lines which were compared to our proposed neural network model. In our research as comparisons we have used classical measures: Simple Moving Average, Trend Line and Exponential Moving Average. These measures are influenced by many factors, such as number of tests carried out in given country or region, restrictions imposed by government, restrictions imposed by state authorities, and finally behavior of the population in given area. Anyway classical measures of statistic are frequently used to make predictions. All applied statistical measures are presented in charts for sample countries and regions in Figs 8 and 7.

Simple Moving Average (SMA) is modeled by equation:

$$\begin{array}{c} SMA=\frac{\sum_{i=1}^{n}c_{i}}{n} \end{array}$$ (6)

where *n* is a number of factors taken into account, *c*_*i*_ is *i*-th value of the considered set.

Exponential Moving Average (EMA) is modeled by equation:

$$\begin{array}{c} EMA_{i}=Y_{i}\cdot a+EMA_{i-1} \end{array}$$ (7)

where *EMA*_0_ = *Y*_0_ and *Y* is *i*-th value of the considered set, $a=\frac{2}{n-1}$ where *n* is the number of values of the considered set.

Trend Line (TL) was built by using equation:

$$\begin{array}{c} \left\{ \begin{array}{c} TL=a\cdot x+b\\ a=\frac{\sum_{i=1}^{n}\left(X_{i}-\bar{X}\right)\cdot \left(Y_{i}-\bar{Y}\right)}{\sum_{i=1}^{n}{\left(X_{i}-\bar{X}\right)}^{2}}\\ b=\bar{Y}-a\cdot \bar{X} \end{array}\right. \end{array}$$ (8)

where *X*_*i*_ and $\bar{X}$ are as follows: *i*-th value of the considered set and arithmetic mean of the considered set *X*, *Y*_*i*_ and $\bar{Y}$ are as follows: *i*-th value of the considered set and arithmetic mean of the set *Y*, *a* is directional coefficient of the straight line, *b* is free expression element.

### 3.5 Numerical results

**Fig 5:**
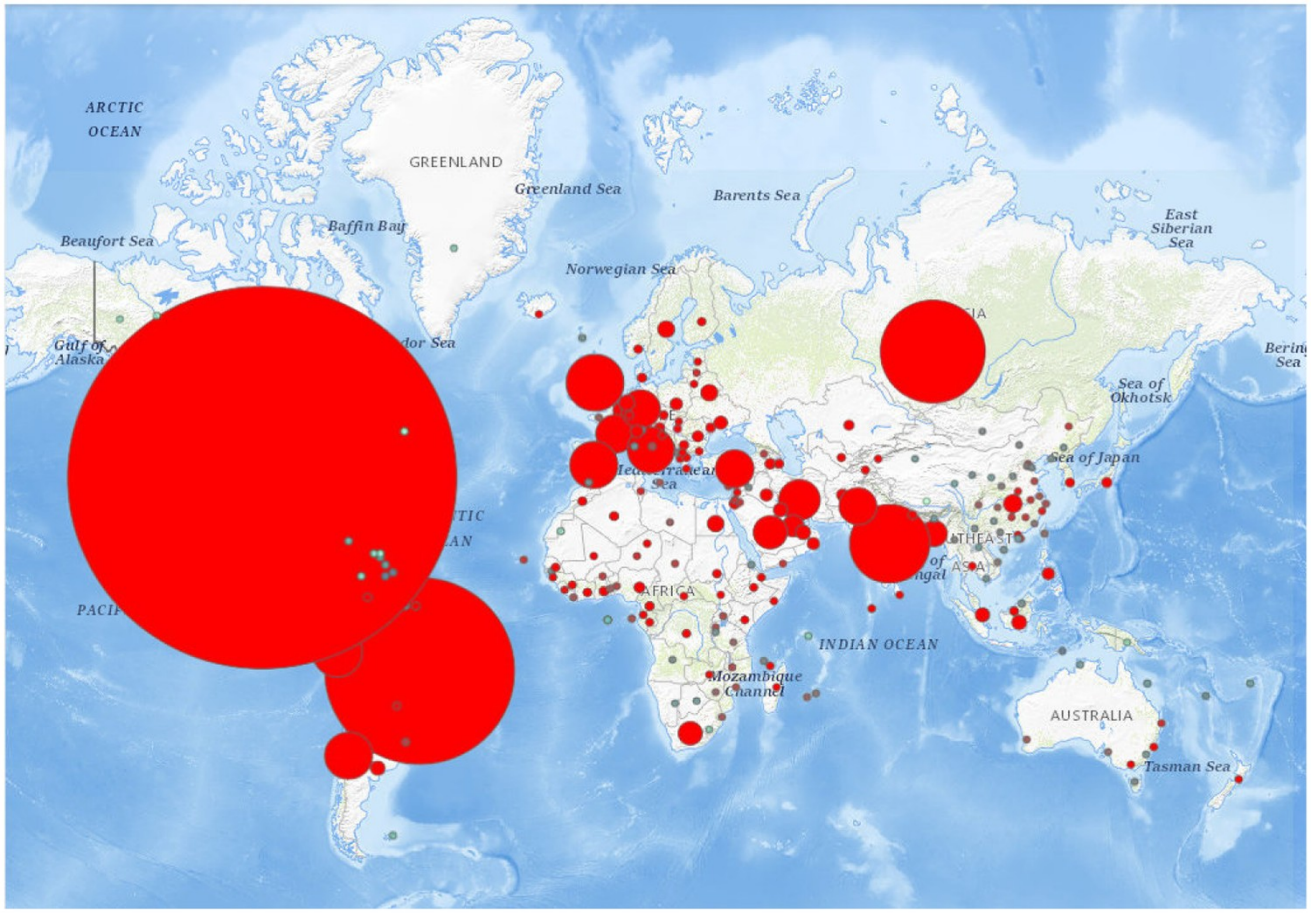
The world map resulted from our system with marked places of prediction. We can see the spots, where the predicted growth is visible in the size of the spot: bigger spot means bigger growth and smaller spot means smaller growth. Color also is related to prediction: the more red spots show places with higher potential growth of cases and the more green spots show places with higher decrease of cases or no cases at all. The map was taken from USGS National Map Viewer (public domain): http://viewer.nationalmap.gov/viewer/ as suggested by PlosONE editors.

**Fig 6:**
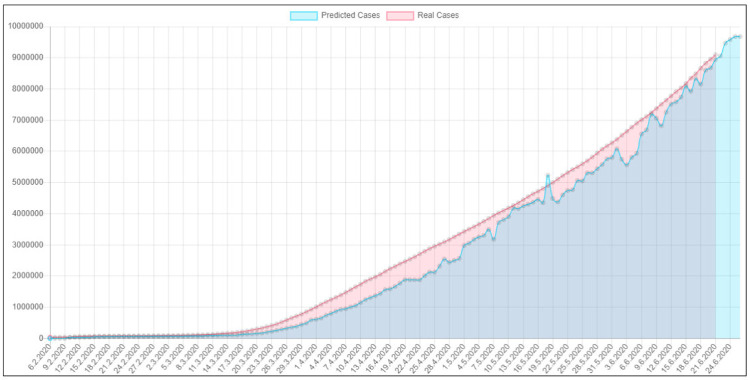
Predicted total number of cases in the world in relation to the real numbers from official governmental services.

In Fig 5 we can see predictions of our model for countries on our planet. The spots are representing predicted trends of cases for world countries. Color red indicates increase, while color green indicates decrease of cases. The bigger the size of the spot the higher growth of cases our system predicts. Fig 6 presents the trend in total number of cases. The results we can see in the image were predicted using data from past weeks, while these results show prediction for next week. Our model was verified in past days, since prediction was correct and the trend of cov19 was correctly predicted by our neural network model we confirmed efficiency of the solution.

**Fig 7:**
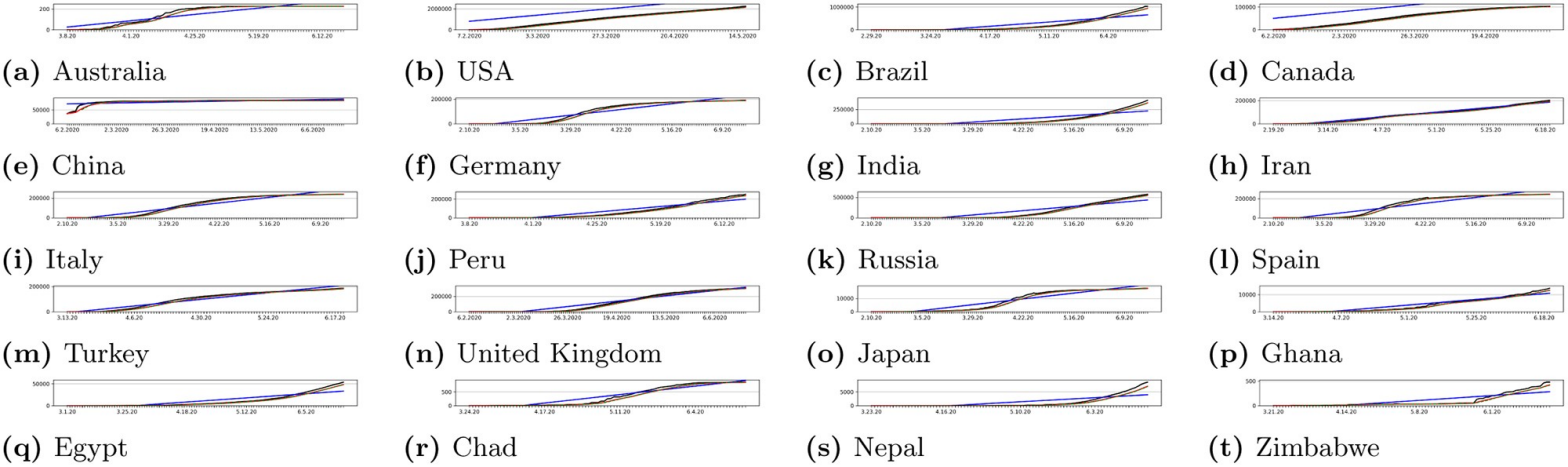
Sample statistical modeling results for some countries. We can see how the classical statistical measures would model predictions of cases. Red line presents real cases, blue line presents Simple Moving Average (SMA), green line presents Exponential Moving Average (EMA) and purple line presents Trend Line (TL).

**Fig 8:**
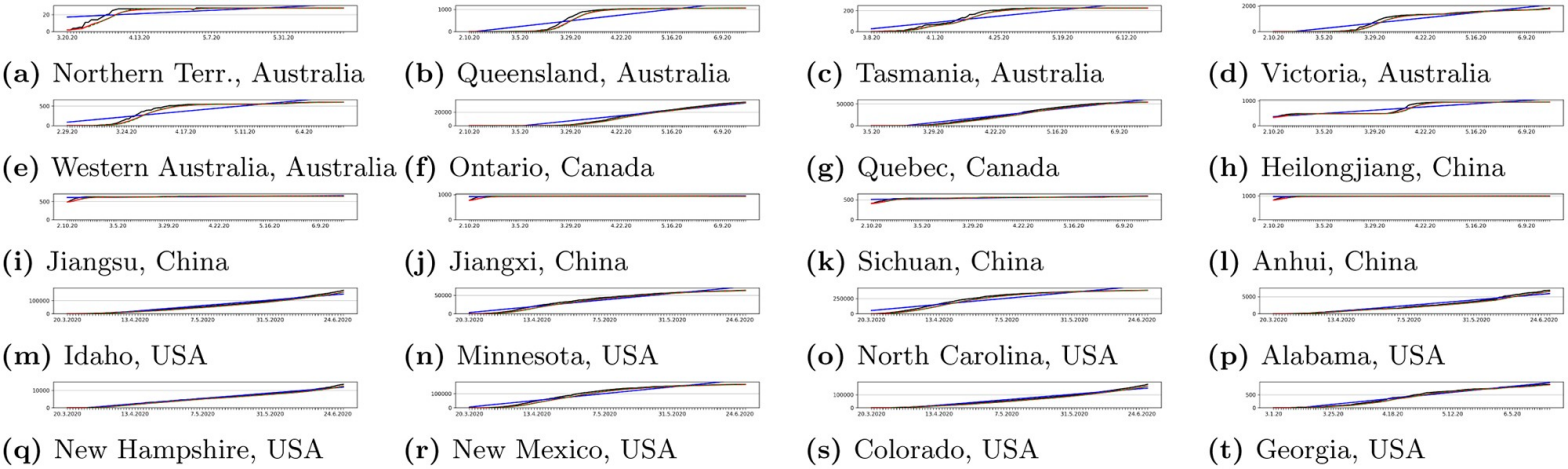
Sample statistical modeling results for some regions from selected countries. We can see how the classical statistical measures would model predictions of cases. Red line presents real cases, blue line presents Simple Moving Average (SMA), green line presents Exponential Moving Average (EMA) and purple line presents Trend Line (TL).

Figs 7 and 8 present results of using statistical models discussed in Section 3.4. Presented results show that applied metrics may have important differences in prediction when compared to official data. Therefore our proposed neural network predictor can solve such problem and reliably predict future trends.

**Fig 9:**
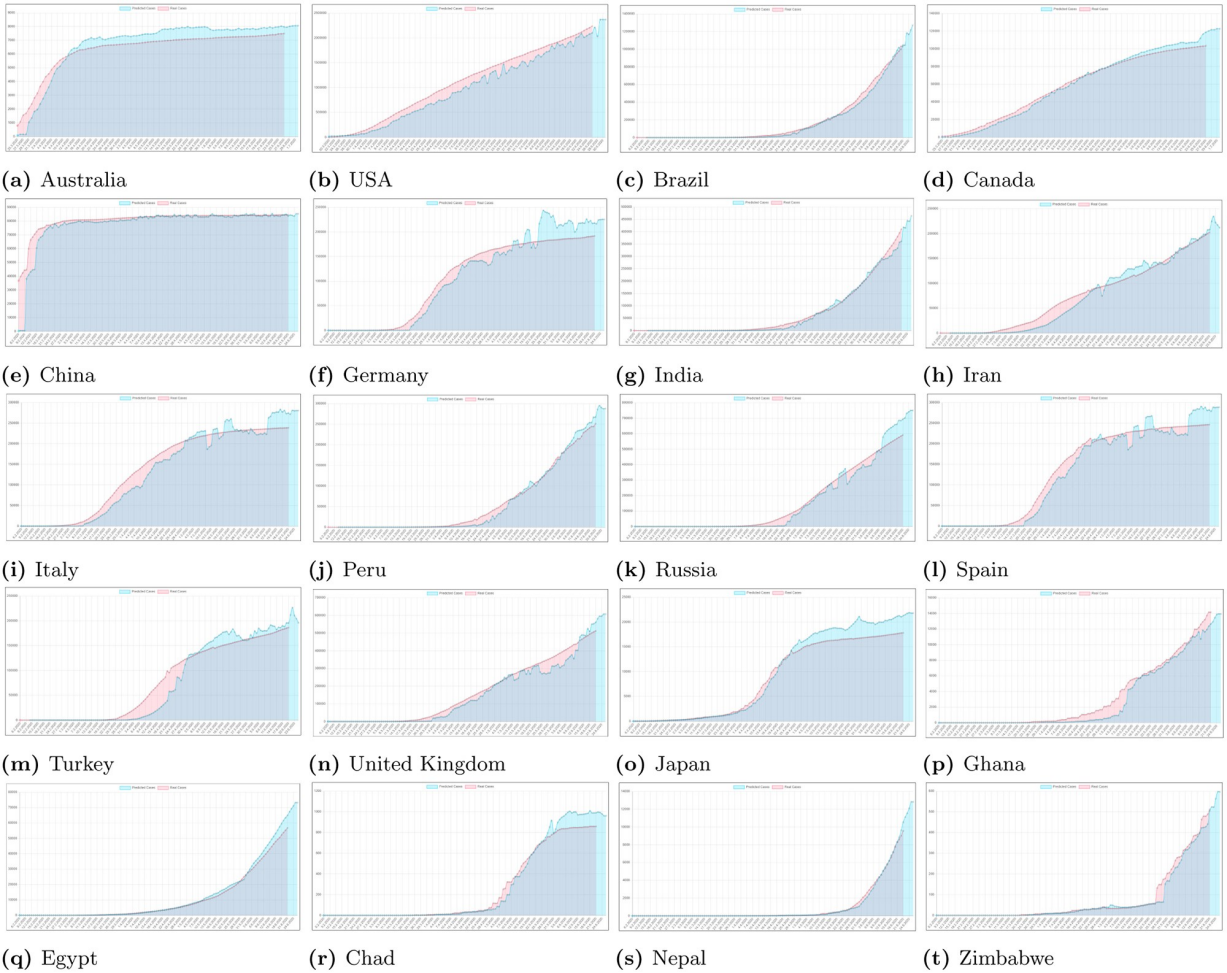
Sample results for some countries. We can see how the proposed model predicts the number of cases in relation to the real number from governmental services.

**Fig 10:**
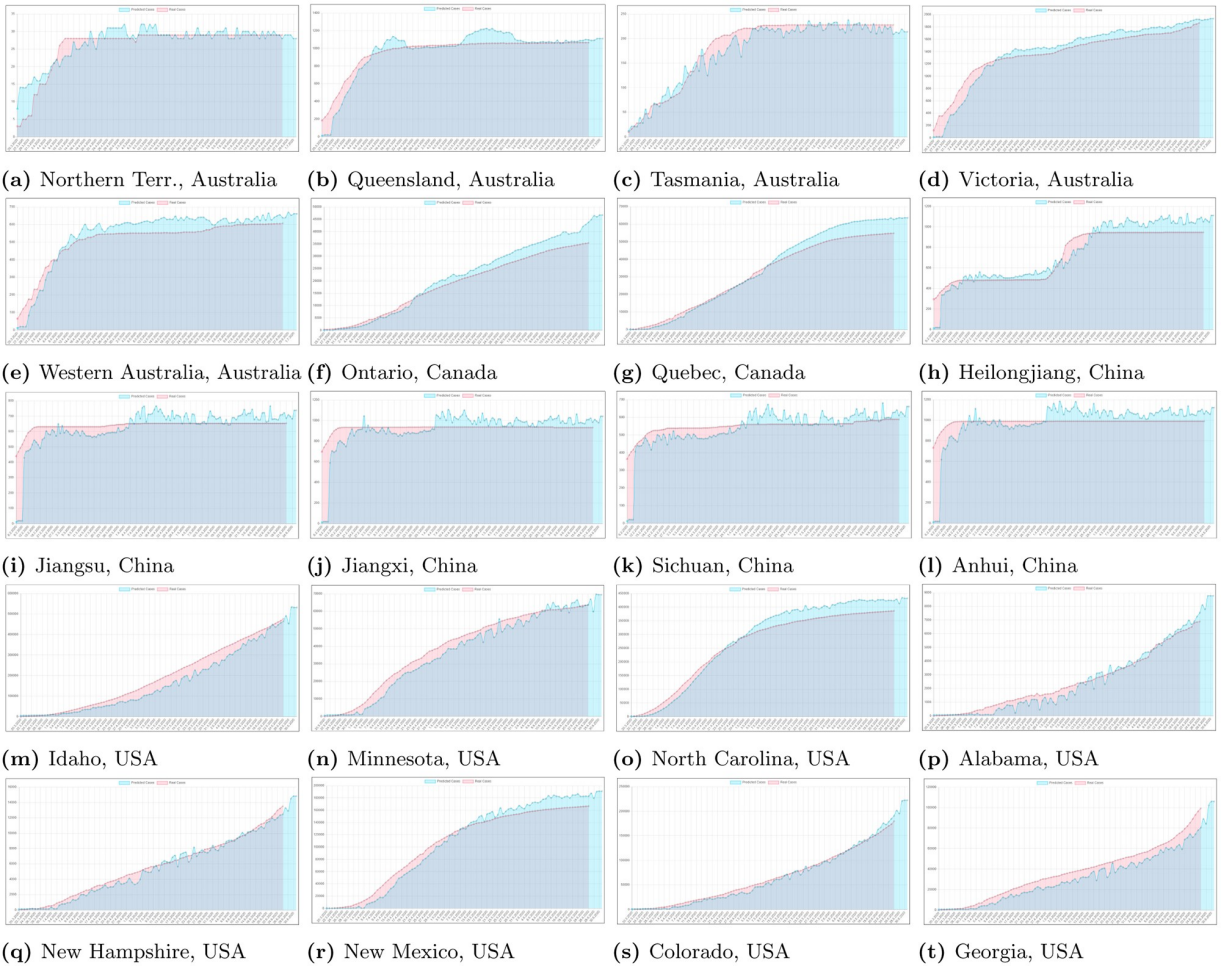
Sample results for some regions of selected countries. We can see how the proposed model predicts the number of cases in relation to the real number from governmental services.

**Fig 11:**
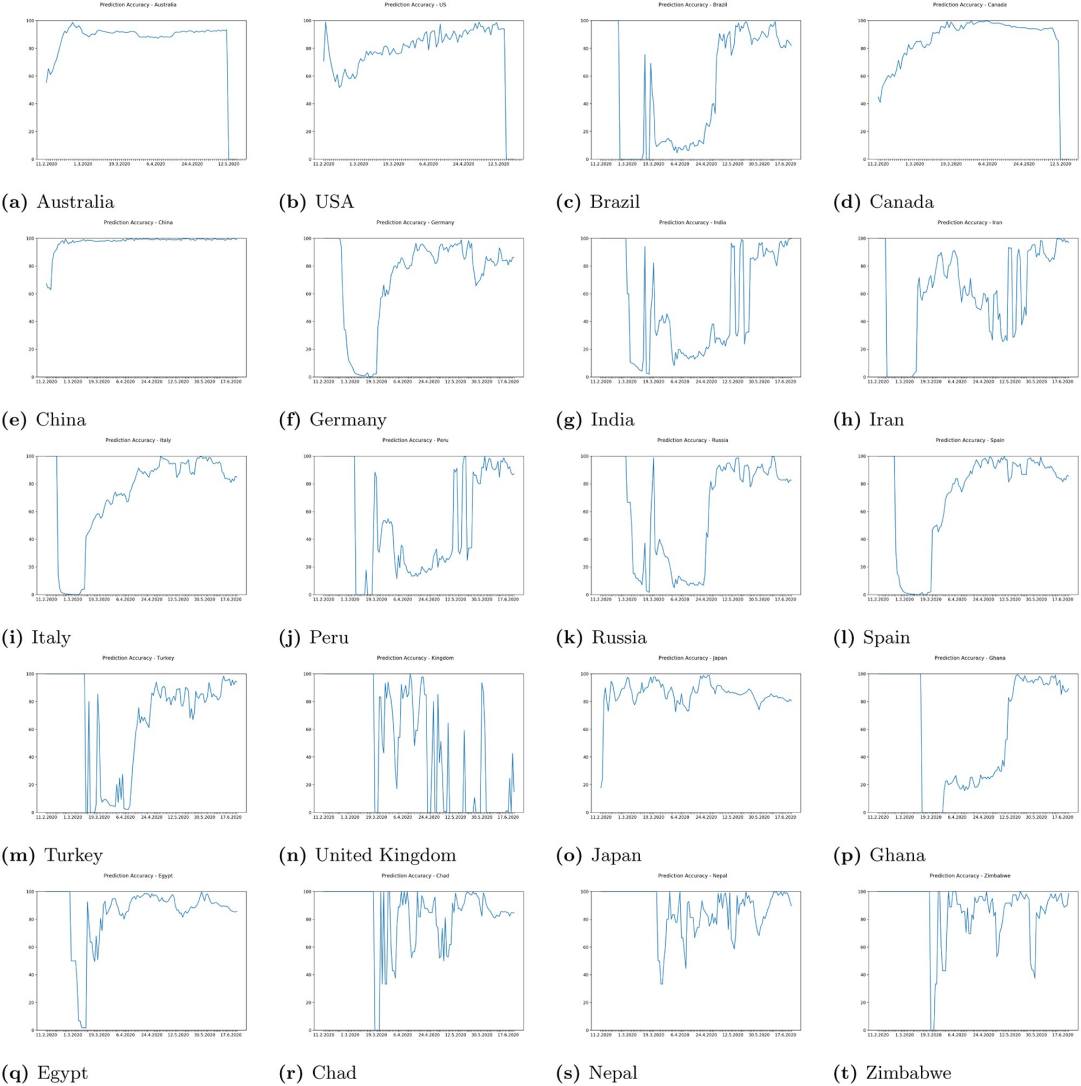
Plots of accuracy per day for some countries with margin 0.2 during days of our experiment.

**Fig 12:**
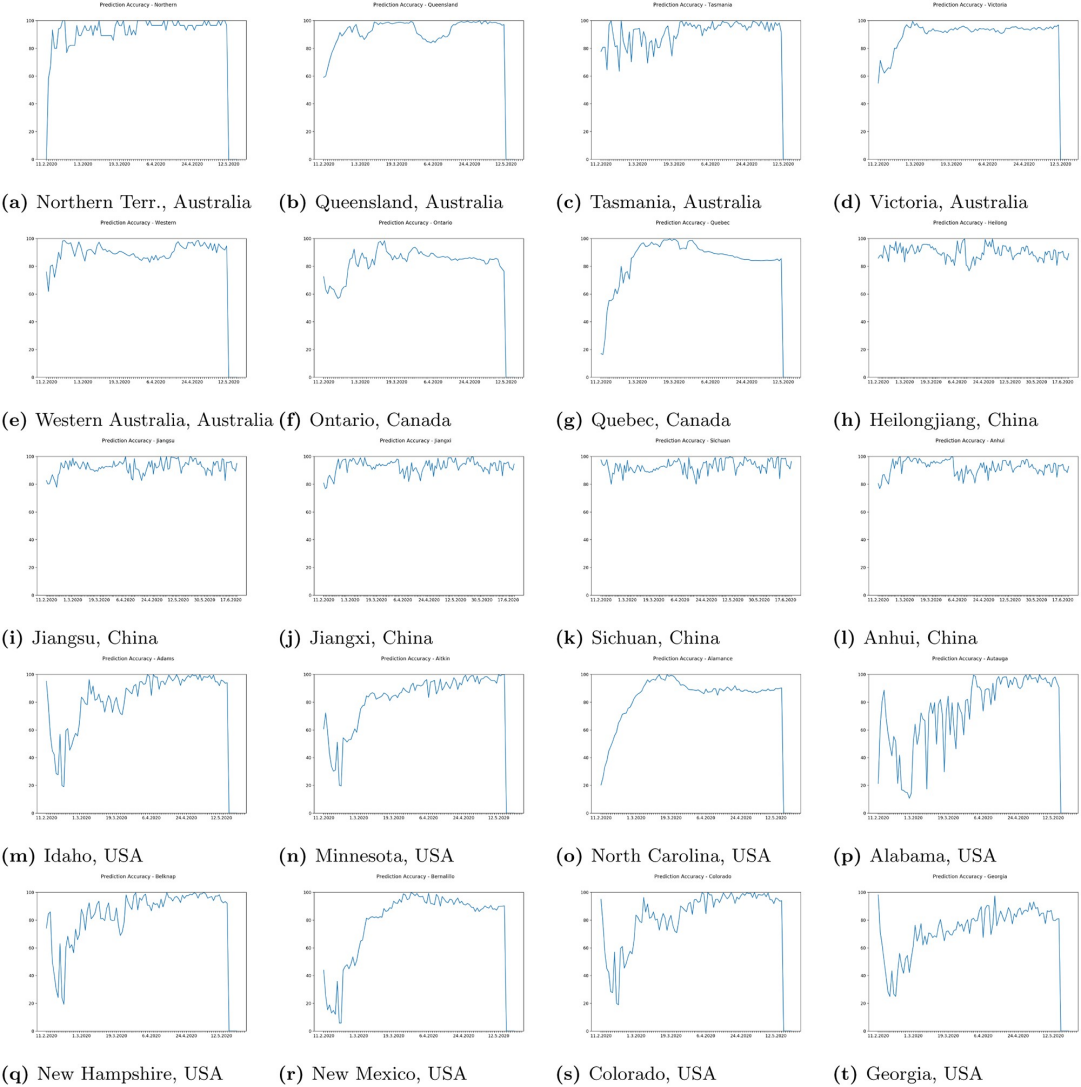
Sample accuracy of our proposed neural network prediction model with different error margins in some regions.

In Figs 9 and 10 we can see some example prediction charts of our model compared to real numbers provided by governmental services worldwide. When we analyze these results we can see that the model presented in Fig 2 works well. Predicted lines closely meet the real numbers for various countries. Similar situation is visible for model presented in Fig 3. Results visible in Fig 10 show how the model predicts the trend in case of regions in USA, Australia, Canada and China. Similarly, results for regions also closely meet the real numbers both in case when the growth is visible or where we encounter stabilization. In each case the prediction curve is closely located to real number curve what shows good efficiency of our model. Figs 11 and 12 show accuracy change for training of our models. In case of some countries and regions fluctuations are visible. These result from rapidly changing values, however the most important for the final efficiency is the accuracy the model receives after training. In Table 2 we can see the final accuracy of our prediction model margin for countries, and in Table 3 for some regions in USA, Australia, Canada and China.

**Table 2:** Sample accuracy of our proposed neural network prediction model with different error margins in some countries.

Error Margin	Brazil	Australia	Canada	China	Germany	India	Iran	Italy	Peru	Russia
0.05	53.49%	92.24%	90.48%	98.83%	74.72%	55.33%	63.23%	75.19%	57.45%	64.32%
0.1	55.28%	94.48%	91.7%	99.07%	77.43%	56.89%	65.71%	77.52%	59.06%	66.48%
0.15	56.57%	94.99%	92.71%	99.25%	79.59%	58.23%	67.85%	79.35%	60.29%	68.04%
0.2	57.42%	95.27%	93.48%	99.42%	81.05%	59.34%	69.71%	80.68%	61.39%	69.22%
0.25	58.03%	95.53%	94.05%	99.58%	81.88%	60.5%	71.66%	81.97%	62.48%	70.11%
0.3	58.58%	95.79%	94.55%	99.77%	82.56%	61.8%	73.66%	83.15%	63.72%	70.95%
0.35	59.17%	96.01%	95.07%	99.96%	83.19%	63.28%	75.63%	84.05%	65.18%	71.79%
0.4	59.84%	96.12%	95.54%	100.0%	83.74%	65.0%	77.5%	84.76%	66.89%	72.64%
0.45	60.63%	96.18%	95.81%	100.0%	84.11%	66.87%	78.82%	85.37%	68.88%	73.62%
0.5	61.57%	96.18%	95.97%	100.0%	84.43%	69.08%	79.97%	85.76%	70.97%	74.76%
Error Margin	Spain	Turkey	United Kingdom	United States	Chad	Egypt	Ghana	Japan	Nepal	Zimbabwe
0.05	74.32%	71.52%	53.59%	86.03%	86.53%	88.16%	62.83%	90.2%	90.53%	89.66%
0.1	76.29%	73.91%	55.63%	88.6%	88.73%	90.5%	63.87%	94.38%	92.75%	91.5%
0.15	77.79%	75.88%	57.77%	90.72%	90.46%	91.87%	64.69%	97.34%	94.58%	92.64%
0.2	78.74%	77.28%	59.67%	92.48%	91.42%	92.51%	65.4%	98.77%	96.07%	93.39%
0.25	79.43%	78.27%	61.44%	93.79%	92.22%	93.01%	66.1%	99.25%	97.11%	93.99%
0.3	79.94%	79.12%	63.12%	94.57%	92.95%	93.52%	66.89%	99.42%	97.85%	94.55%
0.35	80.41%	79.89%	64.71%	95.24%	93.74%	94.05%	67.81%	99.5%	98.4%	94.98%
0.4	80.94%	80.36%	66.24%	95.87%	94.53%	94.5%	68.88%	99.57%	98.81%	95.46%
0.45	81.5%	80.75%	67.68%	96.11%	95.24%	94.97%	70.14%	99.64%	99.13%	95.93%
0.5	82.1%	81.18%	69.02%	96.18%	95.77%	95.47%	71.59%	99.7%	99.46%	96.45%

**Table 3:** Sample accuracy of our proposed neural network prediction model with different error margins in some regions.

Error Margin	Northern Australia, Australia	Queensland, Australia	Tasmania, Australia	Victoria, Australia	Western Australia, Australia	Ontario, Canada	Quebec, Canada	Heilong, China	Jiangsu, China	Jiangxi, China
0.05	93.41%	93.38%	92.86%	93.13%	92.68%	88.1%	88.66%	94.75%	96.74%	97.16%
0.1	94.43%	94.64%	94.18%	94.23%	94.71%	91.11%	90.78%	98.1%	99.03%	99.08%
0.15	94.87%	95.3%	95.04%	94.66%	95.62%	93.33%	92.16%	99.57%	99.73%	99.7%
0.2	95.17%	95.55%	95.61%	95.05%	95.9%	94.17%	92.71%	99.96%	99.98%	99.95%
0.25	95.27%	95.75%	95.9%	95.38%	96.02%	94.73%	93.17%	100.0%	100.0%	100.0%
0.3	95.39%	95.91%	96.08%	95.73%	96.09%	95.25%	93.6%	100.0%	100.0%	100.0%
0.35	95.5%	96.06%	96.17%	96.02%	96.15%	95.81%	94.01%	100.0%	100.0%	100.0%
0.4	95.57%	96.17%	96.18%	96.12%	96.18%	96.11%	94.35%	100.0%	100.0%	100.0%
0.45	95.63%	96.18%	96.18%	96.18%	96.18%	96.18%	94.64%	100.0%	100.0%	100.0%
0.5	95.67%	96.18%	96.18%	96.18%	96.18%	96.18%	94.79%	100.0%	100.0%	100.0%
Error Margin	Sichuan, China	Anhui, China	Idaho, USA	Minnesota, USA	North Carolina, USA	Alabama, USA	New Hampshire, USA	New Mexico, USA	Colorado, USA	Georgia, USA
0.05	97.06%	96.64%	86.57%	86.27%	88.07%	80.4%	87.76%	83.63%	86.57%	79.57%
0.1	99.3%	98.86%	88.15%	88.21%	90.61%	82.29%	89.48%	85.61%	88.15%	82.49%
0.15	99.88%	99.67%	89.54%	89.69%	91.75%	83.8%	90.73%	86.65%	89.54%	85.27%
0.2	100.0%	99.96%	90.67%	90.51%	92.31%	85.31%	91.72%	87.37%	90.67%	87.63%
0.25	100.0%	100.0%	91.42%	91.19%	92.85%	86.58%	92.43%	87.91%	91.42%	89.47%
0.3	100.0%	100.0%	92.07%	91.82%	93.32%	87.75%	93.07%	88.47%	92.07%	90.99%
0.35	100.0%	100.0%	92.7%	92.49%	93.72%	88.7%	93.56%	89.11%	92.7%	91.97%
0.4	100.0%	100.0%	93.35%	93.22%	94.09%	89.61%	93.97%	89.73%	93.35%	92.72%
0.45	100.0%	100.0%	93.9%	93.86%	94.46%	90.48%	94.23%	90.41%	93.9%	93.43%
0.5	100.0%	100.0%	94.3%	94.31%	94.8%	91.22%	94.49%	91.14%	94.3%	94.07%

In our research we have used a measure of error margin to evaluate accuracy of our system. Predicted cases count is categorized as matching:

$$\begin{array}{c} a=\{\begin{array}{c} predicted>real-(real*error\_margin)\\ predicted<real+(real*error\_margin) \end{array} \end{array}$$ (9)

where *a* is match. Because of that our margin is relative to the cases count and allows us to better validate the overall accuracy of our proposed system. Tables 2 and 3 present the final level of accuracy achieved. As we can see the change depends on the level of error margin accepted for the research experiment. The higher the margin the more accurate the system is. Present daily changes of accuracy of our prediction model for selected countries and regions in the World show that the highest decrease in accuracy of our model prediction for world was in the time when cov19 was spreading among continents and where authorities we introducing periodical lockdown, however our model gained accuracy again very fast and adjusted to the rapidly changing situation.

## 4 Discussion

Results of our prediction model show many strong points in the proposed approach. The structure of the developed system gives many advances. Proposed two types of neural architectures are devoted to prediction from small cov19 data so that the results both for countries and regions are well adequate to the real numbers. Applied two types of activation functions gave the neural network ability for exact fitting to the normalized data from various locations. The architecture is trained by ADAM algorithm so that the error rate is low and the system is well developed. The situation is changing fast so we can also see changes in the predicted numbers reflected in our system accuracy. On the other hand the system gains efficiency very fast and learns the new data with good precision. The statistics of the system show that with new incoming data proposed model is better trained to prediction about the situation in each country or region.

Proposed neural network predictor has an important advantages over other approaches. First of them is good adjustment to the new information. When the neural network is trained it easily adopts to new data and gives correct predictions. It is not necessary to analytically model the trend line, which is the key factor for purely mathematical predictors. On the other hand for neural networks well training a reasonable amount of data on the input is necessary to achieve good accuracy. However as we have shown in our model we can simulate these by architecture of the neural network and data preprocessing. Due to the nature of incoming information in this case we have developed two architectures for countries and regions, but each of them is fed with different number of values from previous days. The interpretation of our results is the trend of the situation (growing or decreasing) and potential number of new cases. For both of them our neural network predictor works well.

## 5 Conclusions and future works

We have presented developed system for prediction of cov19 spread. Applied neural network architectures give good results and help to show the growth trend for countries and regions. The situation of any disease or epidemia is changing very fast so it is hard for any mathematical model to perfectly fit to the real numbers. On the other hand prediction models sourced in Artificial Intelligence give flexibility to the changing situation to match new incoming data while maintaining accuracy in prediction. Therefore such models can be used to reliably estimate changes in uncertain environments by using predicted tends of growth. The hards thing in case of cov19 prediction is that the project started with very limited number of information about cases and additionally the situation was changing rapidly from day to day. Therefore in such conditions it is very hard to model and train the neural network to achieve reliable results. Although such conditions our model proved to work well. Applied data selection on the input, normalization and proposed division made it possible for our proposed architectures to train well for predictions in countries and regions.

Our future works will be oriented to introduce some technique for automatic adjustment of the neural network to newly incoming data. We think that transfer learning may help in that case, ei. when similar region or country have similar values of recorded numbers so that procedures of transfer learning maybe help in development of prediction system. Especially such procedure may be important when in the beginning of projects there is a little number of information available.
